# Optimal adaptive barrier-function super-twisting nonlinear global sliding mode scheme for trajectory tracking of parallel robots

**DOI:** 10.1016/j.heliyon.2023.e13378

**Published:** 2023-02-02

**Authors:** Mostafa Barghandan, Ali Akbar Pirmohamadi, Saleh Mobayen, Afef Fekih

**Affiliations:** a Department of Mechanical Engineering, University of Zanjan, Zanjan, Iran; b Department of Electrical Engineering, University of Zanjan, Zanjan, Iran; c Graduate School of Intelligent Data Science, National Yunlin University of Science and Technology, Douliou, Yunlin, Taiwan; d Department of Electrical and Computer Engineering, University of Louisiana at Lafayette, Lafayette, USA

**Keywords:** Trajectory tracking, Sliding mode control, Barrier function, Super-twisting approach, Adaptive control

## Abstract

Compared to serial robots, parallel robots have potential superiorities in rigidity, accuracy, and ability to carry heavy loads. On the other hand, the existence of complex dynamics and uncertainties makes the accurate control of parallel robots challenging. This work proposes an optimal adaptive barrier-function-based super-twisting sliding mode control scheme based on genetic algorithms and global nonlinear sliding surface for the trajectory tracking control of parallel robots with highly-complex dynamics in the presence of uncertainties and external disturbances. The globality of the proposed controller guarantees the elimination of the reaching phase and the existence of the sliding mode around the surface right from the initial instance. Moreover, the barrier-function based adaptation law removes the requirement to know the upper bounds of the external disturbances, thus making it more suitable for practical implementations. The performance and efficiency of the controller is assessed using simulation study of a Stewart manipulator and an experimental evaluation on a 5-bar parallel robot. The obtained results were further compared to that of a six-channel PID controller and an adaptive sliding mode control method. The obtained results confirmed the superior tracking performance and robustness of the proposed approach.

## 1 Introduction

Parallel manipulators, also called parallel robots or parallel kinematic machines (PKM), are defined as manipulators which end-effector movement is controlled by at least two kinematic chains connected from the end-effector to the fixed base [[1], [2], [3], [4]]. Compared to serial robots, parallel robots have potential superiorities in rigidity, accuracy, and ability to carry heavy loads [5]. There are many usages for parallel robots in several fields, such as motion simulators, haptic devices, micro-mechanisms and high precision machine tools [6,7], agriculture [8], industry [9], medical research [10] sectors, and also high-speed pick-and-place (PnP) applications in industrial fields [11], such as pharmacy, auto, food industries and electronics [12]. The primary problem lies in finding a solution to their coupled, and complex nonlinear dynamic models which not only can sufficiently characterize the real robotic system but also can be calculated in real-time for execution into a control scheme [13], which needs an extremely advanced control method to attain the expected dynamic efficiency. In addition, such robots are often subject to a variety of uncertainties (time-varying or constant), caused by external disturbances, unknown dynamics, nonlinear friction forces, and unknown nonlinear terms of the used dynamic model. Those uncertainties have the potential to compromise the performance of the controller [14]. Hence, the precise motion control of parallel robots is challenging problem, especially considering the possible uncertainties in real-time applications [15]. In the past decades, researchers have investigated the performance improvement of the position control of parallel robots, which require more complicated control algorithms than that of serial robots.

Various trajectory tracking and position control approaches, such as the augmented proportional derivative (APD) control [16], nonlinear proportional-integral-derivative (PID) control [17] have been proposed in the literature for parallel robots. One of the most significant drawbacks of these methods is the lack of controller robustness. To overcome this problem and improve the robustness properties of the parallel robots controller, various advanced methods have been suggested in the literature. Examples are the asymptotic output-tracking control [18], adaptive control [19], adaptive output-feedback control [20], and adaptive regulation control based on approximation [21]. Moreover, a robust nonlinear controller (RNC), which is the combination of nonlinear PD control with robust dynamics compensation, is proposed in Ref. [22] to eliminate the effects of unmodeled dynamics, nonlinear friction, and external disturbances. Though the above-mentioned advanced control methods can improve the trajectory tracking performance, those controllers rely on the availability of a relatively accurate and complete dynamic model and are computationally expensive, which limit their practical implementation to parallel manipulators. Additionally, the uncertainties and neglected dynamics may lead to the instability of the closed-loop system with a nominal controller; accordingly, it is necessary to use a robust approach such as sliding mode control (SMC). There have been much literature about the dynamics of the Stewart manipulator (SM). Some simplified dynamic models that omit the effects of the friction and leg dynamics can be found in Refs. [23,24]. The simplified leg models have been improved in Refs. [25,26]. A more general model that includes the viscous friction of the joints based on the Newton–Euler method has been proposed in Ref. [27]. The Lagrange technique has been utilized in Ref. [28]. Some other techniques have also been suggested, such the screw theory [28], generalized momentum method [29], virtual work principle [30], and Kane's equations [31]. The control of such robots is subject to time-varying or constant uncertainties, which can be affected by unknown uncertainties, friction forces, unknown nonlinear dynamics, and probabilistic external disturbances. Suchlike uncertainties may weaken the efficiency of the systems controller [32].

SMC, as a robust control method is a method that has a good efficiency against plant uncertainties, nonlinearities, external disturbances and time-varying parameters [[33], [34], [35], [36], [37], [38]]. The global SMC (GSMC) technique was proposed to remove the reaching phase such that the switching phase exists right from the beginning [[39], [40], [41]]; however, it is necessary to know the initial conditions of the sliding surface, which is possible and easy to obtain. On the other hand, the forenamed SMC and GSMC methods are fulfilled in infinite time because of the asymptotic convergence of the linear sliding surface. Therefore, via the linear switching surfaces, the control signal may have an unsatisfactory convergence speed in finite time [42]. In order to overcome this problem, finite-time control schemes have been pursued for robotic systems based on the finite-time stability concept [43]. The terminal sliding mode control (TSMC) method has been designed to force the state trajectories to reach the equilibrium point in finite time [44,45]. TSMC is appropriate for high-precision control and also provides fast response as it accelerates the convergence rate around the equilibrium point; therefore TSMC is designed based on the nonlinear sliding surfaces to attain finite-time stability without the cost of a large control signal [46,47]. Also, in Ref. [48], the high-speed nonsingular terminal switched sliding mode control technique is proposed for the control of robotic manipulators. In another research, the integral terminal sliding mode cooperative control of multi-robot networks is investigated [49]. The composite integral terminal sliding mode-based adaptive synchronization control of multiple robotic manipulators with actuator saturation is proposed in Ref. [50]. Additional sliding mode-based control and adaptive control techniques can further be found in the literature [[51], [52], [53]]. However, none of the researches [54,55] have been focused on the adaptive barrier-function-based TSMC approach for development of robust tracking control of nonlinear perturbed systems.

In the present paper, we propose a novel optimal adaptive barrier-function super-twisting global nonlinear sliding mode controller for the trajectory tracking applications of parallel robots. The control signal is generated based on Lyapunov stability theory, and implemented to the Stewart manipulators, a six degrees of freedom (6-DOF) parallel robots with complicated dynamics, under external disturbances and model uncertainties. The main contributions of this work are summarized as follows:

(1) A control technique that eliminates the reaching mode and thereby guarantee system stability from its initial state thanks to its global property
(2) A realistic design that considers an adaptation law to eliminate the need to know any information about the upper bounds of external disturbances; thus, making it more suitable for practical implementations.
(3) A control approach with a minimum number of optimally tuned parameters using genetic algorithms.

The rest of this paper is organized as follows. Sect. 2 states the problem description and provides some preliminaries. Sect. 3 discusses the proposed control method for the high precision control of the parallel manipulator, and derives the proof of system using the Lyapunov stability criteria. Sect. 4, provides the simulation results aiming at assessing the efficiency and advantages of the suggested method in comparison with some conventional and nonlinear control methods. Finally, some conclusions are given in Sect. 5. s.

## 2 Dynamic model of the parallel robot

**Fig. 1:**
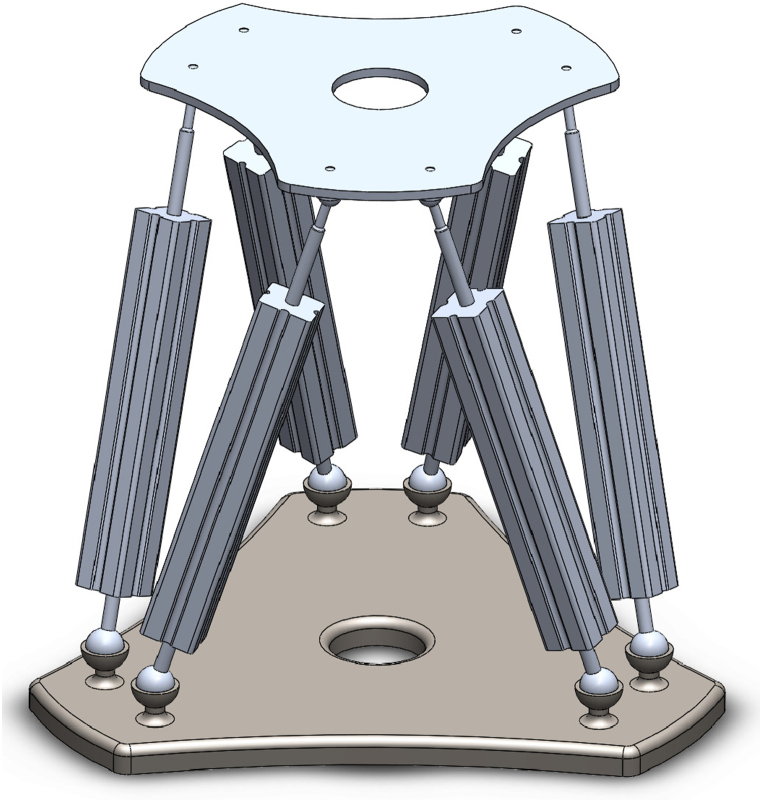
Design of 6-SPS type of Stewart manipulator (Stewart platform) modeled in SolidWorks 2021 SP4.

**Fig. 2:**
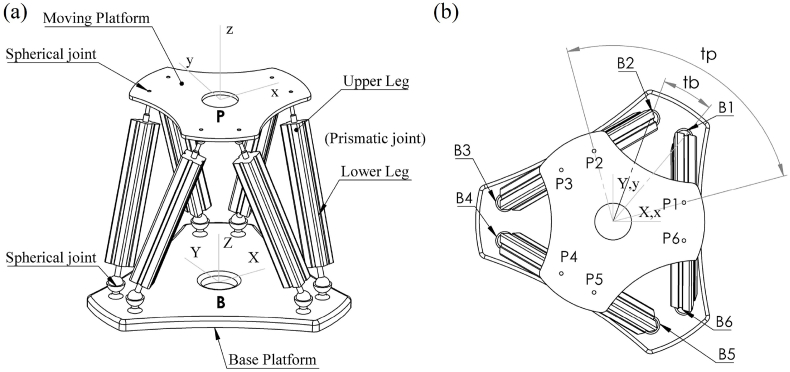
Schematic diagram of 6-SPS Stewart Manipulator (Hexapod). (a) Isometric view (b) Top view.

In this section, the dynamics of Stewart manipulator are formulated. SM is a parallel manipulator with three translational and three rotational DOFs, which consists of six prismatic legs that are connected by two plates, as shown in Fig. 1. The schematic view of a parallel robot is illustrated in Fig. 2. One of the mentioned plates is the base platform (BP) which is fixed in space, and the second plate is named as moving platform (MP), which is moving with six DOF; the remaining six prismatic joints (legs) are linked to the BP and MP by universal or spherical joints which are called 6-UPU, 6-UPS, 6-SPU, and 6-SPS manipulator types. For example, in the 6-SPU mechanism, the prismatic legs are connected to the BP and MP by spherical and universal joints, respectively. Then, the designed controller for this manipulator can be easily implemented to other types of parallel mechanisms. As shown in Fig. 2, the inertial coordinate (IC) system *B*_*XYZ*_ is located at the BP with its origin at the geometry center of BP, the moving frame (MF) $P_{XYZ}$ is fixed to the MP center of mass. The three translational DOFs in this robot are translations on direction of the *X, Y, Z* axes, and the three rotational DOFs are the rotations about the axis *BX, BY* and, *BZ*.

Define the displacement and orientation vector as:

(1) $$q={\left[x,y,z,\alpha ,\beta ,\gamma \right]}^{T}$$

where (x,y,z) denote the mass origin linear motions of MP with respect to IC, and (*α*, *β*, *γ*) represent the rotational motions with respect to *X*-axis, *Y*-axis, and *Z*-axis, individually.

Consider the model of the dynamic equation of 6-DOF SM as [28,56]:

(2) $$M\left(q\right)\ddot{q}+V\left(q,\dot{q}\right)\dot{q}+G\left(q\right)+F_{d}\dot{q}+F_{s}\left(\dot{q}\right)+\tau_{d}\left(q,\dot{q}\right)=J^{T}\left(q\right)\tau$$

where q is defined previously in Eq. (1); $M\left(q\right)\in R^{6\times 6}$ is the inertia matrix; $V\left(q,\dot{q}\right)\dot{q}\in R^{6\times 1}$ is the Coriolis and centrifugal force vector; *G*(*q*) is the six-dimensional gravitational force vector; $F_{s}\left(\dot{q}\right)\in R^{6\times 1}$ is the static friction vector; $F_{d}\in R^{6\times 6}$ is the dynamic friction coefficient matrix; $J\left(q\right)\in R^{6\times 6}$ is the Jacobian matrix; $\tau \in R^{6\times 1}$ is the input forces vector from prismatic leg's actuator; $\tau_{d}$ is the 6 × 1 vector of bounded input force disturbance with $\left\|\tau_{d}\right\|\leq d$, where *d* is a positive scalar; *M*(*q*), *G*(*q*) and $V\left(q,\dot{q}\right)$ can be written as

(3) $$M\left(q\right)=M_{0}\left(q\right)+\Delta M\left(q\right)$$

(4) $$V\left(q,\dot{q}\right)=V_{0}\left(q,\dot{q}\right)+\Delta V\left(q,\dot{q}\right)$$

(5) $$G\left(q\right)=G_{0}\left(q\right)+\Delta G\left(q\right)$$

where $M_{0}\left(q\right)$ in Eq. (3), $V_{0}\left(q,\dot{q}\right)$ in Eq. (4), and $G_{0}\left(q\right)$ in Eq. (5) are the nominal parts (See Eq. (45), Eq. (46), Eq. (47) in Appendix A), and ΔM(q), $\Delta V\left(q,\dot{q}\right)$, and ΔG(q) are the uncertain bounded parts of M(q), $V\left(q,\dot{q}\right)$, and G(q). The nominal parts are defined in Appendix A, which appears at the end of the article.

The Jacobian matrix for dynamics (2) is described as:

(6) $$J=\left[\begin{array}{c} \begin{array}{ccc} {\vec{L}}_{1} & {\vec{L}}_{2}\; & \begin{array}{ccc} \begin{array}{cc} {\vec{L}}_{3}\; & {\vec{L}}_{4}\; \end{array} & {\vec{L}}_{5} & {\vec{L}}_{6} \end{array} \end{array}\\ \begin{array}{ccc} \begin{array}{ccc} \begin{array}{cc} R_{P}^{B}S_{1} & R_{P}^{B}S_{2} \end{array} & R_{P}^{B}S_{3} & R_{P}^{B}S_{4} \end{array} & R_{P}^{B}S_{5} & R_{P}^{B}S_{6} \end{array} \end{array}\right],R_{P}^{B}=R_{\alpha }R_{\beta }R_{\gamma },S_{i}={\vec{P}}_{i}\times {\vec{L}}_{i}$$

where ${\vec{L}}_{i}$’s are the leg's vectors on the fixed-base frame; $P_{i}$’s are the upper joints positions on the moving platform frame; $R_{\alpha }=\left[\begin{array}{ccc} 1 & 0 & 0\\ 0 & \cos\;\alpha & \sin\;\alpha \\ 0 & -\sin\;\alpha & \cos\;\alpha \end{array}\right]$, $R_{\beta }=\left[\begin{array}{ccc} \cos\;\beta & 0 & -\sin\;\beta \\ 0 & 1 & 0\\ \sin\;\beta & 0 & \cos\;\beta \end{array}\right]$, $R_{\gamma }=\left[\begin{array}{ccc} \cos\;\gamma & \sin\;\gamma & 0\\ -\sin\;\gamma & \cos\;\gamma & 0\\ 0 & 0 & 1 \end{array}\right]$ in Eq. (6) are the standard rotation matrices about X, Y and Z axis, respectively. Then, Eq. (2) can be represented as:

(7) $$M\left(q\right)\ddot{q}+\Psi \left(q,\dot{q}\right)+F_{d}\dot{q}+F_{s}\left(\dot{q}\right)+\tau_{d}\left(q,\dot{q}\right)=u\left(t\right)$$

where $u\left(t\right)=J^{T}\left(q\right)\tau$ is the control input; $\Psi \left(q,\dot{q}\right)=V\left(q,\dot{q}\right)\dot{q}+G\left(q\right)$. Then, in the presence of the uncertainties and external disturbances, it can be considered as $\Psi \left(q,\dot{q}\right)=\Psi_{0}\left(q,\dot{q}\right)+\Delta \Psi \left(q,\dot{q}\right)$, where $\Psi_{0}\left(q,\dot{q}\right)$ and $\Delta \Psi \left(q,\dot{q}\right)$ are the nominal and unknown parts of $\Psi \left(q,\dot{q}\right)$, correspondingly. Then, the dynamical equation (7) can be derived as follows:

(8) $$\ddot{q}=-{M_{0}}^{-1}\left(q\right)\left(\Psi_{0}\left(q,\dot{q}\right)+F_{d}\dot{q}+F_{s}\left(\dot{q}\right)+\tau_{d}\left(q,\dot{q}\right)+\Delta \Psi \left(q,\dot{q}\right)+\Delta M\left(q\right)\ddot{q}-u\left(t\right)\right)$$

From Eq. (8), the dynamics of the system can be simplified as below:

(9) $$\ddot{q}={M_{0}}^{-1}\left(q\right)u\left(t\right)+\Pi \left(q,\dot{q}\right)+\Lambda_{0}\left(t\right)$$

where $\Pi \left(t\right)=-{M_{0}}^{-1}\left(q\right)\left\{F_{d}\dot{q}+F_{s}\left(\dot{q}\right)+\tau_{d}\left(q,\dot{q}\right)+\Delta \Psi \left(q,\dot{q}\right)+\Delta M\left(q\right)\ddot{q}\right\}$ is the disturbance and lumped uncertainty as unknown part of system dynamics and in Eq. (9), $\Lambda_{0}\left(t\right)=-{M_{0}}^{-1}\left(q\right)\Psi_{0}\left(q,\dot{q}\right)$ is the bounded known nonlinear part of system dynamics.

Lemma 1 [57]: *Assume that there exists a continuous and positive-definite functional* V(t) *which fulfills a differential equation for* $t\geq t_{0}$ *and* $V\left(t_{0}\right)\geq 0$ *as*:

(10) $$\dot{V}\left(t\right)\leq -\alpha V\left(t\right)-\beta V{\left(t\right)}^{\eta }\text{,}$$

*in* Eq. (10), α,β>0,0<η<1. *Then*, *the functional* V(t) *converges to the origin in finite time* ($t_{S}$) *as* Eq. (11):

(11) $$t_{S}\leq t_{0}+\frac{1}{\alpha \left(1-\eta \right)}\ln\frac{\alpha V{\left(t_{0}\right)}^{1-\eta }+\beta }{\beta }\text{.}$$

Lemma 2 [58]: *Suppose that the continuous positive-definite* V(t) *gratifies the differential inequality for every* $t\geq t_{0}$ *and* $V\left(t_{0}\right)\geq 0$ *as follows*:

(12) $$\dot{V}\left(t\right)\leq \beta V{\left(t\right)}^{\eta }\text{,}$$

*in* Eq. (12) α,β>0,0<η<1 *are two constants*. *Then*, *the functional* V(t) *converges to the origin in the finite time as* Eq. (13):

(13) $$t_{S}\leq t_{0}+\frac{V{\left(t_{0}\right)}^{1-\eta }}{\beta \left(1-\eta \right)}\text{.}$$

## 3 Main results

In the SMC design procedure, the sliding surface selection remarkably affects the system's tracking performance. The sliding manifold is designed in such a way that when it reaches the origin; as a result, the system can obtain the anticipated performance. Let:

(14) $$e\left(t\right)=q-q_{d}\;\dot{e}\left(t\right)=\dot{q}-{\dot{q}}_{d}$$

where e(t) and $\dot{e}\left(t\right)$ are the trajectory tracking errors and their derivatives; $q_{d}$ represents the desired trajectory; ${\dot{q}}_{d}$ denotes the desired trajectory derivative.

### 3.1 Stability analysis and controller design

**Fig. 3:**
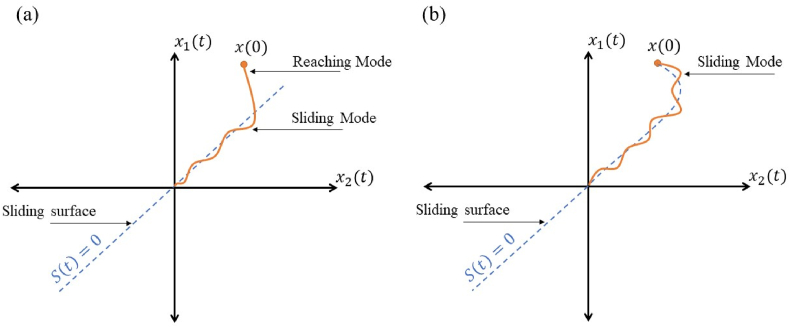
The concept of sliding mode control. (a) Sliding mode method (b) Global sliding mode method.

In order to achieve a sliding mode control (Fig. 3-a) with a nonlinear sliding surface for the dynamic system (9), the sliding surface can be proposed as:

(15) $$s\left(t\right)=\dot{e}\left(t\right)+\lambda e\left(t\right)$$

where λ is the positive coefficient. Then, the global sliding manifold (Fig. 3-b) is configured as Eq. (16) to attain a robust global nonlinear sliding surface, which eliminates the reaching mode and subsequently leads to the existence of the error states on the sliding surface right from the initial instant:

(16) ∂(t)=Ω(s(t)−s(0)exp(−εt))

where Ω is a constant row vector, and ε is the positive value.

Remark 1 Compared with the sliding manifold $\hat{\partial }\left(t\right)=\Omega \left(\dot{e}\left(t\right)-e\left(0\right)\exp\left(-\varepsilon t\right)\right)$, the nonlinear global sliding manifold (Eq. (16)) forces the error dynamics to reach the surface from the initial instance. Accordingly, the robust behavior of the system in the attendance of disturbances is guaranteed.

If ∂(t)=0, then from Eq. (16), it can be written:

(17) $$\hat{\partial }\left(t\right)=\hat{\partial }\left(0\right)\exp\left(-\varepsilon t\right)$$

It can be seen that Eq. (17) is the solution of the first-order differential equation (Eq. (18)):

(18) $$\dot{\hat{\partial }}\left(t\right)+\varepsilon \;\hat{\partial }\left(t\right)=0$$

The goal of the GSMC law is for the error trajectory e(t) to achieve the sliding surface from the starting instance and move on the sliding surface to the equilibrium point. By taking the time-derivate of Eq. (16), we have:

(19) $$\dot{\partial }\left(t\right)=\Omega \left(\dot{s}\left(t\right)+\varepsilon s\left(0\right)\exp\left(-\varepsilon t\right)\right)=\Omega \left(\ddot{e}\left(t\right)+\lambda \dot{e}\left(t\right)+\varepsilon s\left(0\right)\exp(-\varepsilon t\right)$$

where considering $\ddot{e}\left(t\right)=\ddot{q}-{\ddot{q}}_{d}\text{,}$ it is obtainable from Eq. (14) and Eq. (19) that:

(20) $$\dot{\partial }\left(t\right)=\Omega \left(\ddot{q}-{\ddot{q}}_{d}+\lambda \dot{e}\left(t\right)+\varepsilon s\left(0\right)\exp(-\varepsilon t\right)=\Omega \left(\Lambda_{0}\left(q,\dot{q}\right)-{\ddot{q}}_{d}+\lambda \dot{e}\left(t\right)+\varepsilon s\left(0\right)\exp\left(-\varepsilon t\right)+M_{0}{\left(q\right)}^{-1}u\left(t\right)+\Pi \left(t\right)\right)$$

One can obtain the equivalent control law from Eq. (21) for the error dynamics, $\dot{\partial }\left(t\right)=0$ is the necessary condition to stay on the sliding manifold ∂(t), while in order to achieve the equivalent control law, the system uncertainties and external disturbances Π(t) are not taken into account. Afterward, the equivalent control is obtained as:

(21) $$u_{eq}\left(t\right)=-M_{0}\left(q\right)\left(\Lambda_{0}\left(q,\dot{q}\right)-{\ddot{q}}_{d}+\lambda \dot{e}\left(t\right)+\varepsilon s\left(0\right)\exp\left(-\varepsilon t\right)\right)\text{.}$$

However, there is no guarantee for satisfactory control performance if the system external disturbances and uncertainties Π(t) are considered. Hence, to remove the effects of the unwanted perturbations, an auxiliary control law should be defined. In practical terms, there is no exact information about the upper bound of system perturbations, and therefore, the term ∥Π(t)∥ is not easy to be characterized. Suppose that the unknown perturbations are bounded, i.e., >∥Π(t)∥ , where Γ is a positive unknown constant. Besides, assume that $\hat{\Gamma }$ is an estimation value for Γ, that is obtained by the following adaptive law:

(22) $$\dot{\hat{\Gamma }}=\kappa ∥\partial \left(t\right)\Omega^{T}∥$$

where κ is a constant and positive value. Then, the auxiliary control law expression can be stated as

(23) $$u_{aux}\left(t\right)=-M_{0}\left(q\right)\left(\hat{\Gamma }sgn\left(\partial \left(t\right)\Omega^{T}+k\partial \left(t\right)\Omega^{T}\right)\right)$$

where k is a constant and positive value. The first part of Eq. (23) is an adaptive control law to compensate for the bounded uncertainties. The second part in Eq. (23) is a proportional control law toward the sliding manifold that is used as a feedback term to augment the system's stability and ameliorate the transient response.

By integrating Eq. (22) from the dynamics of the sliding manifold ∂(t), one can obtain an estimation of the parameter Γ as:

(24) $$\hat{\Gamma }=k\int ∥\partial \left(t\right)\Omega^{T}∥dt\text{.}$$

Substituting Eq. (24) into Eq. (23) yields the following proportional-integral control law:

(25) $$u_{aux}\left(t\right)=-M_{0}\left(q\right)\left(k\partial \left(t\right)\Omega^{T}+\kappa sgn\left(\partial \left(t\right)\Omega^{T}\right)\int \left\|\partial \left(t\right)\Omega^{T}\right\|dt\right)=-M_{0}\left(q\right)sgn\left(\partial \left(t\right)\Omega^{T}\right)\left(k\left\|\partial \left(t\right)\Omega^{T}\right\|+\kappa \int \left\|\partial \left(t\right)\Omega^{T}\right\|dt\right)=k_{p}\left\|\partial \left(t\right)\Omega^{T}\right\|+k_{i}\int \left\|\partial \left(t\right)\Omega^{T}\right\|dt\text{,}$$

where $k_{p}=-kM_{0}\left(q\right)sgn\left(\partial \left(t\right)\Omega^{T}\right)$ and $k_{i}=-\kappa M_{0}\left(q\right)\times sgn\left(\partial \left(t\right)\Omega^{T}\right)$. The determination of the design parameters $k_{p}$ and $k_{i}$ is dependent on the case. The multiplier k and κ parameters in Eq. (25) should be designed to ensure that the system states converge to the desired value. The phrase $\kappa \int \left\|\partial \left(t\right)\Omega^{T}\right\|dt$ is considered as an integral control input which is an approximator for disturbances in order to eliminate the steady-state error. By enhancing the values of κ, the steady-state error is decreased and the adaptation law becomes faster. In order to improve the system stability and dynamic behavior of a proportional control technique, the phrase $k\left\|\partial \left(t\right)\Omega^{T}\right\|$ is used. Furthermore, this term eliminates the disturbance estimation error. For tracking error reduction, large values of k are used; however, this can lead to an increase in overshoot. Vice versa, reducing the value of k results in a small control gain and reduces the accuracy of the trajectory tracking. The complete control law can be achieved from Eq. (21) and Eq. (23) as:

(26) $$u\left(t\right)=u_{eq}\left(t\right)+u_{aux}\left(t\right)=-M_{0}\left(q\right)\left(\Lambda_{0}\left(q,\dot{q}\right)-{\ddot{q}}_{d}+\lambda \dot{e}\left(t\right)+\varepsilon s\left(0\right)\exp(-\varepsilon t\right)+k\partial (t)\Omega^{T}+\hat{\Gamma }sgn(\partial (t)\Omega^{T}))\text{.}$$

Theorem 1 *Consider the nonlinear global sliding manifold defined in* Eq. (8) *and suppose that the external disturbances are bounded and unknown*, *i*.*e*., >‖Π(t)‖ , *where* Γ *is a positive and unknown value*. *Suppose that* $\hat{\Gamma }$ *is the estimation of* Γ *that is obtained by the adaptive law* (Eq. (22)). *By implementation of the adaptive control approach* (Eq. (26)), *the system trajectories* (Eq. (7)) *are converged to the sliding manifold* ∂=0 *and remain on it afterwards*.

*Proof*: *By substituting the control approach* (26) *into* Eq. (20), *one can obtain*:

(27) $$\dot{\partial }\left(t\right)=\Omega \left(-k\partial \left(t\right)\Omega^{T}-\hat{\Gamma }sgn\left(\partial \left(t\right)\Omega^{T}\right)+\Pi \left(t\right)\right)\text{.}$$

*To assess the stability property of the proposed control approach*, *we consider the Lyapunov stability theory*. *Let us choose the following positive-definite equation as a Lyapunov function candidate*:

(28) $$\nu \left(t\right)=\frac{1}{2}\partial {\left(t\right)}^{2}+\frac{1}{2}\gamma \;{\tilde{\Gamma }}^{2}\text{,}$$

*in* Eq. (28), $\tilde{\Gamma }=\hat{\Gamma }-\Gamma \text{.}$ *Taking the time-derivative from* ν(t) *using* Eq. (22) *and* Eq. (27) *yields*:

(29) $$\dot{\nu }\left(t\right)=\partial \left(t\right)\dot{\partial }\left(t\right)+\gamma \;\tilde{\Gamma }\dot{\tilde{\Gamma }}=\partial \left(t\right)\Omega \left(\Pi \left(t\right)-k\partial \left(t\right)\Omega^{T}-\hat{\Gamma }sgn\left(\partial \left(t\right)\Omega^{T}\right)\right)+\gamma \left(\hat{\Gamma }-\Gamma \right)\dot{\hat{\Gamma }}=\partial \left(t\right)\Omega \left(\Pi \left(t\right)-k\partial \left(t\right)\Omega^{T}-\hat{\Gamma }sgn\left(\partial \left(t\right)\Omega^{T}\right)\right)+\gamma \kappa \left(\hat{\Gamma }-\Gamma \right)\left\|\partial \left(t\right)\Omega^{T}\right\|\leq \left\|\partial \left(t\right)\Omega \right\|\left\|\Pi \left(t\right)\right\|-\hat{\Gamma }\left\|\partial \left(t\right)\Omega \right\|+\gamma \kappa \left\|\partial \left(t\right)\Omega \right\|\left(\hat{\Gamma }-\Gamma \right)+\left\|\partial \left(t\right)\Omega \right\|\Gamma -\left\|\partial \left(t\right)\Omega \right\|\Gamma =-\left\|\partial \left(t\right)\Omega \right\|\left(\Gamma -\left\|\Pi \left(t\right)\right\|\right)-\left(1-\gamma \kappa \right)\left\|\partial \left(t\right)\Omega \right\|\left(\hat{\Gamma }-\Gamma \right)\text{.}$$

*since* Γ>‖Π(t)‖ *and* γκ<1 , *hence* Eq. (29) *can be stated by*:

(30) $$\dot{\nu }\left(t\right)\leq -\sqrt{2}\left\|\Omega \right\|\left(\Gamma -\left\|\Pi \left(t\right)\right\|\right)\frac{\left|\partial \left(t\right)\right|}{\sqrt{2}}-\sqrt{\frac{2}{\gamma }}\left(1-\gamma \kappa \right)\frac{\tilde{\Gamma }}{\sqrt{\frac{2}{\gamma }}}\left\|\partial \left(t\right)\Omega \right\|$$

$$\leq -\min\left\{\sqrt{2}\left\|\Omega \right\|\left(-\left\|\Pi \left(t\right)\right\|\right),\sqrt{\frac{2}{\gamma }}\left(-\gamma \kappa \right)\left\|\partial \left(t\right)\Omega \right\|\right\}\times \left(\frac{\left|\partial \left(t\right)\right|}{\sqrt{2}}+\frac{\tilde{\Gamma }}{\sqrt{\frac{2}{\gamma }}}\right)=-I\nu {\left(t\right)}^{\frac{1}{2}}\text{,}$$

*where* $I=\min\left\{\sqrt{2}\left\|\Omega \right\|\left(\Gamma -\left\|\Pi \left(t\right)\right\|\right),\sqrt{\frac{2}{\gamma }}\left(1-\gamma \kappa \right)\left\|\partial {\left(t\right)}^{T}\Omega \right\|\right\}>0$. *Eventually*, *by taking advantage of the tuning law using the adaptive controller*, *the nonlinear global sliding manifold is converged to zero in finite time*. *□*

Remark 2 *In the proposed design method*, *the sliding surfaces*∂(t)*are converged to zero in finite time* (*according to the proof of* Theorem 1 *in* Eq. (30)); *however*, *because of the definition of global sliding surface* (Eq. (16)), *the tracking error trajectories* (Eq. (14)) *are converged to the origin asymptotically*.

Remark 3 *In order to avoid the chattering phenomena*, *the following modification to the adaptive control approach* (26) *can be carried out*:

(31) $$u\left(t\right)=-M_{0}\left(q\right)\left(\Lambda_{0}\left(q,\dot{q}\right)-{\ddot{q}}_{d}+\lambda \dot{e}\left(t\right)+\varepsilon s\left(0\right)\exp\left(-\varepsilon t\right)+k\partial \left(t\right)\Omega^{T}+\hat{\Gamma }sat\left(\frac{\partial \left(t\right)\Omega^{T}}{\delta }\right)\right)$$

*where the operator sat* (.) *acts as a saturation function with the boundary layer thickness equal to δ*.

Remark 4 *Due to the use of the saturation function in* Eq. (31), *it is not possible for the sliding manifold* Eq. (16) *to be equal to zero for all the times*. *Thereupon*, *the adaptive parameters will be incremented steadily and boundlessly*. *To overcome this issue*, *the* Eq. (22) *can be rectified as* Eq. (32):

(32) $$\dot{\hat{\Gamma }}=\left\{ \begin{array}{c} 0\;if\;\left\|\partial \left(t\right)\Omega^{T}\right\|\leq \delta \\ \kappa \left\|\partial \left(t\right)\Omega^{T}\right\|\;if\;\left\|\partial \left(t\right)\Omega^{T}\right\|>\delta \end{array}\right.$$

*Although the well-known technique to circumvent the chattering phenomenon uses a continuous saturation function instead of a discontinuous sign function*; *the implementation of such boundary layer technique is abolished* [59]. *A thin boundary layer may not eliminate the chattering phenomenon*, *and a wide boundary layer leads to a steady-state error*. *A super-twisting procedure is an appropriate substitute for the saturation function for the chattering phenomena avoidance without affecting the trajectory tracking performance* [60,61].

*The super-twisting GSMC scheme can be adjusted as*

(33) $$u\left(t\right)=-M_{0}\left(q\right)\left(\Lambda_{0}\left(q,\dot{q}\right)-{\ddot{q}}_{d}+\lambda \dot{e}\left(t\right)+\varepsilon s\left(0\right)\exp\left(-\varepsilon t\right)+k\partial \left(t\right)\Omega^{T}+\mu_{0}{\left|\partial \left(t\right)\Omega^{T}\right|}^{0.5}sgn\left(\partial \left(t\right)\Omega^{T}\right)+\upsilon_{a}\right),{\dot{\upsilon }}_{a}=\hat{\Gamma }sgn\left(\partial \left(t\right)\Omega^{T}\right)\text{.}$$

*where*$\mu_{0}$*is a positive and constant value*.

*The proposed method in* Eq. (33) *includes a combination of two sections GSMC and a super-twisting algorithm in which the super twisting idea presented in* [[61], [62], [63]] *has been used*.

Remark 5 *In the presented adaptation law*, *the adaptation gain may grow with a very slight slope*. *Although this is not reflected in the provided simulations*, *it can be problematic in the long run*. *In order to overcome this issue*, *the planned method of* [64] *is suggested*, *which consists of a dynamic adaptive control gain establishing the sliding mode in a finite time*.

Remark 6 *For extending the suggested control technique*, *the barrier function adaptive sliding mode controller is investigated for robust tracking control of dynamics of parallel robot in the presence of external disturbances*. *A novel adaptive control input based on barrier function is designed in this section*. *The disturbance terms can be estimated using the barrier function adaptive controller more proficiently*, *and the closed-loop system becomes more stable*. *Using the control law* Eq. (26) *with*:

(34) $$\hat{\Gamma }\left(t\right)=\left\{ \begin{array}{c} {\hat{\Gamma }}_{a}\left(t\right),\text{if}\;0<t\leq \bar{t}\\ {\hat{\Gamma }}_{psb}\left(t\right),\text{if}\;t>\bar{t} \end{array}\right.$$

*in* Eq. (34), $\bar{t}$ *is the time that the tracking error trajectories converge to the neighborhood* ε *of surface* ∂(t). *The adaptation control law and positive-semi-definite* (*PSD*) *barrier function are determined by* Eq. (35) *and* Eq. (36):

(35) $${\dot{\hat{\Gamma }}}_{a}\left(t\right)=\psi {\left|\partial \left(t\right)\right|}^{\frac{m}{n}-1}$$

(36) $${\hat{\Gamma }}_{psb}\left(t\right)=\frac{\left|\partial \left(t\right)\right|}{\varepsilon -\left|\partial \left(t\right)\right|}$$

*where* ε *is a positive coefficient* [65]. *Employing the adaptation law* Eq. (35), *the controller gain is tuned to increase until error signals reach the neighborhood* ε *at time* $\bar{t}$. *For times bigger than* $\bar{t}$, *the adaptation gain switches to the PSD barrier function which decreases the convergence region and maintains the errors in that convergence region*. *For the condition* $0<t\leq \bar{t}$, *the controller is proposed in* Theorem 1. *For the other condition when* $t>\bar{t}$, *the barrier function adaptive control law is considered by*:

(37) $$u\left(t\right)=-M_{0}\left(q\right)\left(\Lambda_{0}\left(q,\dot{q}\right)-{\ddot{q}}_{d}+\lambda \dot{e}\left(t\right)+\varepsilon s\left(0\right)\exp(-\varepsilon t\right)+k\partial (t)\Omega^{T}+{\hat{\Gamma }}_{psb}\left(t\right)sgn(\partial (t)\Omega^{T}))\text{.}$$

*The error states reach the region*|∂(t)|≤ε*in finite time*. *The Lyapunov candidate function is constructed by*:

(38) $$w\left(t\right)=0.5\left({\partial \left(t\right)}^{2}+{{\hat{\Gamma }}_{psb}\left(t\right)}^{2}\right)$$

*Time-derivate of* Eq. (38) *is found as*

(39) $$\dot{w}\left(t\right)=\partial \left(t\right)\dot{\partial }\left(t\right)+{\hat{\Gamma }}_{psb}\left(t\right){\dot{\hat{\Gamma }}}_{psb}\left(t\right)$$

*where substituting* $\dot{\partial }\left(t\right)$ *from* Eq. (20) *into* Eq. (39), *one has*:

(40) $$\dot{w}\left(t\right)=\partial \left(t\right)\left(\;\Omega \left(\Lambda_{0}\left(q,\dot{q}\right)-{\ddot{q}}_{d}+\lambda \dot{e}\left(t\right)+\varepsilon s\left(0\right)\exp\left(-\varepsilon t\right)+M_{0}{\left(q\right)}^{-1}u\left(t\right)+\Pi \left(t\right)\right)\right)+{\hat{\Gamma }}_{psb}\left(t\right){\dot{\hat{\Gamma }}}_{psb}\left(t\right)$$

*Now*, *using* Eq. (37) *in the* Eq. (40), *one obtains*:

(41) $$\dot{w}\left(t\right)=\partial \left(t\right)(-{\hat{\Gamma }}_{psb}\left(t\right)sgn\left(\partial \left(t\right)\Omega^{T}\right)+\Pi \left(t\right))+{\hat{\Gamma }}_{psb}\left(t\right){\dot{\hat{\Gamma }}}_{psb}\left(t\right)\leq \left|\partial \left(t\right)\right|\left\{\left|\Pi \left(t\right)\right|-{\hat{\Gamma }}_{psb}\left(t\right)\right\}+{\hat{\Gamma }}_{psb}\left(t\right){\dot{\hat{\Gamma }}}_{psb}\left(t\right)\leq \left|\partial \left(t\right)\right|\left\{\left|\Pi \left(t\right)\right|-{\hat{\Gamma }}_{psb}\left(t\right)\right\}+{\hat{\Gamma }}_{psb}\left(t\right)\frac{\varepsilon }{{\left(\varepsilon -\left|\partial \left(t\right)\right|\right)}^{2}}sgn\left(\partial \left(t\right)\right)\dot{\partial }\left(t\right)\leq \left|\partial \left(t\right)\right|\left\{\left|\Pi \left(t\right)\right|-{\hat{\Gamma }}_{psb}\left(t\right)\right\}+{\hat{\Gamma }}_{psb}\left(t\right)\frac{\varepsilon }{{\left(\varepsilon -\left|\partial \left(t\right)\right|\right)}^{2}}\left[\Pi \left(t\right)-{\hat{\Gamma }}_{psb}\left(t\right)sgn\left(\partial \left(t\right)\right)\right]sgn\left(\partial \left(t\right)\right)$$

Eq. (41) *is rewritten as*:

(42) $$\dot{w}\left(t\right)\leq -\left\{{\hat{\Gamma }}_{psb}\left(t\right)-\left|\Pi \left(t\right)\right|\right\}\left|\partial \left(t\right)\right|-{\hat{\Gamma }}_{psb}\left(t\right)\frac{\varepsilon }{{\left(\varepsilon -\left|\partial \left(t\right)\right|\right)}^{2}}\left[{\hat{\Gamma }}_{psb}\left(t\right)-\left|\Pi \left(t\right)\right|\right]$$

*in* Eq. (42), *because* ${\hat{\Gamma }}_{psb}\left(t\right)\geq \left|\Pi \left(t\right)\right|$ *and* $\frac{\varepsilon }{{\left(\varepsilon -\left|\partial \left(t\right)\right|\right)}^{2}}>0$, *we have*:

(43) $$\dot{w}\left(t\right)\leq -\sqrt{2}\left\{{\hat{\Gamma }}_{psb}\left(t\right)-\left|\Pi \left(t\right)\right|\right\}\frac{\left|\partial \left(t\right)\right|}{\sqrt{2}}-\frac{\sqrt{2}\varepsilon }{{\left(\varepsilon -\left|\partial \left(t\right)\right|\right)}^{2}}\left[{\hat{\Gamma }}_{psb}\left(t\right)-\left|\Pi \left(t\right)\right|\right]\frac{{\hat{\Gamma }}_{psb}\left(t\right)}{\sqrt{2}}\leq -Η\left(\frac{\left|\partial \left(t\right)\right|}{\sqrt{2}}+\frac{{\hat{\Gamma }}_{psb}\left(t\right)}{\sqrt{2}}\right)\leq -Η\;{w\left(t\right)}^{1/2}$$

*in* Eq. (43), $Η=\sqrt{2}\left\{{\hat{\Gamma }}_{psb}\left(t\right)-\left|\Pi \left(t\right)\right|\right\}\min\left\{1,\frac{\sqrt{2}\varepsilon }{{\left(\varepsilon -\left|\partial \left(t\right)\right|\right)}^{2}}\right\}$. *□*

### 3.2 Optimization of controller parameters

In the proposed controller, there are several constants which appropriate choice can directly affect the controller's performance. For the higher performance and accuracy, genetic algorithms are applied for tuning the controller gains and parameters. The fitness function of the genetic algorithm is described as:

(44) $$\xi =\int_{0}^{t}\sqrt{w_{1}{\left(q_{1}-q_{d_{1}}\right)}^{2}+w_{2}{\left(q_{2}-q_{d_{2}}\right)}^{2}+\ldots +w_{i}{\left(q_{i}-q_{d_{i}}\right)}^{2}}dt=\int_{0}^{t}\sqrt{w_{1}{e_{1}}^{2}+w_{2}{e_{2}}^{2}+\ldots +w_{i}{e_{i}}^{2}}$$

where $w_{i}$ is the weighting factor. The goal of the optimization is to minimize the fitness function Eq. (44), thereby minimizing the tracking error of the controlled system.

## 4 Simulation results

**Fig. 4:**
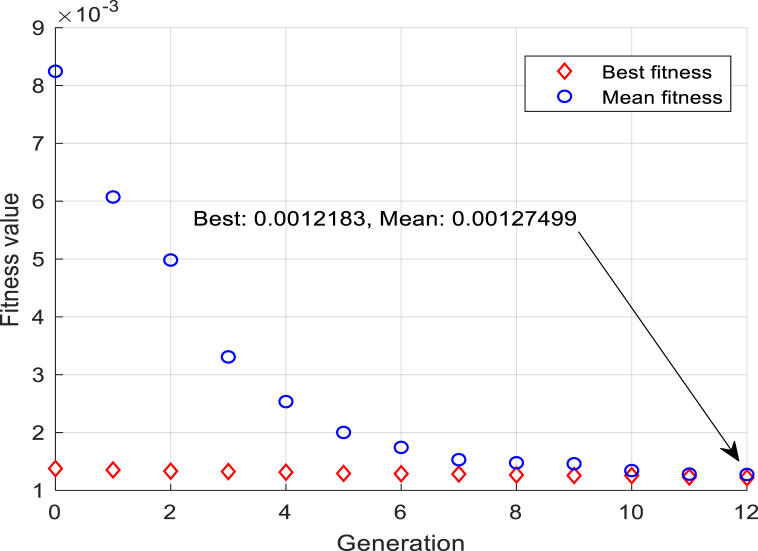
Genetic algorithm diagram: tuning fitness value to reach generations with the best fitness.

In this part, without losing generality, the optimal adaptive barrier-function-based super-twisting nonlinear global sliding mode control is applied to a parallel robot based on Stewart platform Fig. 1, which system dynamics are mentioned in Eq. (2), and the performance of the control is compared with the results of an adaptive nonlinear sliding mode control (ANSMC) and a six-channel PID control method. The geometry and static characteristics of the SM containing mass, mass moment of inertia, and other specifications are provided in Appendix B (Table 3). The genetic algorithm optimization that is implemented with fitness function Eq. (44), illustrated in Fig. 4 which, represents the best value of fitness function reached from 0.001378 to 0.001218, and the mean value converges to the best value in twelve generations. Eventually, the resulted parameters of the proposed controller are listed in Table 1.

**Table 1:** Control gains of the proposed controller for the SM system.

Symbol	Description	Value
λ	Constant Eq. (15)	69.03
Ω	Constant vector Eq. (16)	[1.65, 1.02, 1.91, 1.44, 1.21, 1.96]
ε	Coefficients Eq. (16)	15.34
κ	Coefficients Eq. (22)	2.14
k	Gain Eq. (23)	4.36
$\mu_{0}$	Constant Eq. (33)	0.78

**Table 2:** Control parameters of the proposed controller for the 5-bar robot system.

Symbol	Description	Value
λ	Constant Eq. (15)	35.23
Ω	Constant vector Eq. (16)	[0.91, 1.12]
ε	Coefficients Eq. (16)	10.96
$\hat{\Gamma }$	Coefficients Eq. (22)	3.00
k	Gain Eq. (23)	2.03
$\mu_{0}$	Constant Eq. (33)	0.65

The measurement noise is one of the parameters that has made the difference between simulation and practical implementation. Therefore, to make the results as accurate as possible, Gaussian noise with variance equal to 0.0005, a mean value of zero, and a sampling time equal to 5 ms is considered.

**Fig. 5:**
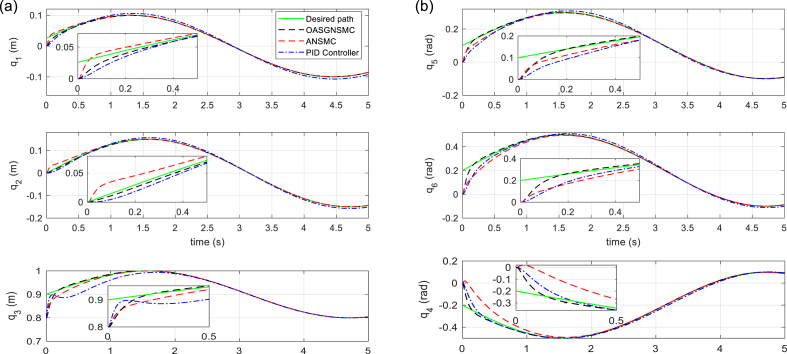
Tracking performance of Stewart Manipulator. (a) Positions (*q*_1_, *q*_2_, *q*_3_) (b) Orientations (*q*_4_, *q*_5_, *q*_6_).

**Fig. 6:**
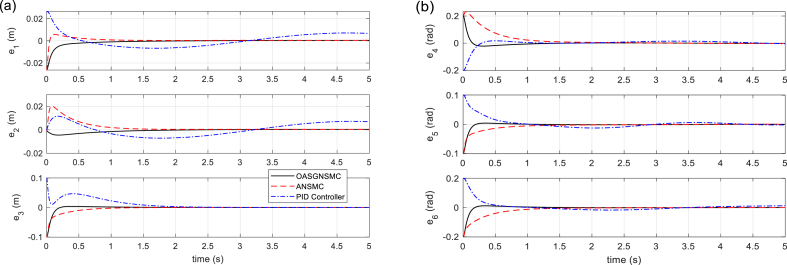
Tracking error of system states. (a) Position errors (*e*_1_, *e*_2_, *e*_3_) (b) Orientation errors (*e*_4_, *e*_5_, *e*_6_).

**Fig. 7:**
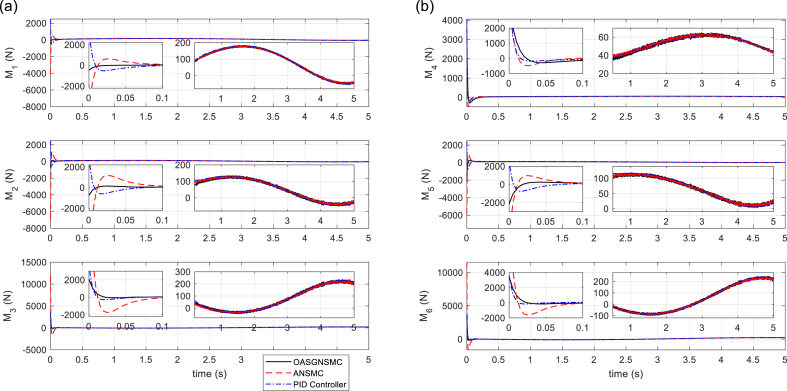
Actuator forces of Stewart prismatic joints. (a) Forces of Joints (*M*_1_, *M*_2_, *M*_3_) (b) Force of Joints (*M*_4_, *M*_5_, *M*_6_).

**Fig. 8:**
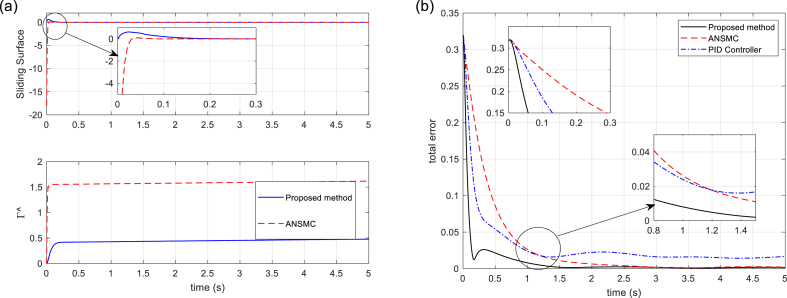
(a) Sliding surface and adaptation gain. (b) Total error signals of controllers.

The tracking performances of SM (displacement and orientation of MP), and tracking errors are illustrated in Fig. 5, Fig. 6, respectively. It is shown that the proposed control technique leads to better tracking, higher accuracy, and faster response than the compared methods. In Fig. 7, the six linear actuator forces are illustrated, which represents the better performance of the proposed control method. On the other hand, it can be concluded that by using the proposed control method, there is slight chattering in the time histories of the control signals, and that is because of the sensor noise. Fig. 8 (left) shows the time responses of the sliding surface and adaptation gain. As it is seen, the sliding surface of the proposed method starts from zero which confirms the globality of the proposed technique. Finally, in Fig. 8 (right), the total error signals of the three controllers are compared. Note that the tracking performance of the proposed approach outperforms that of the other approaches.

## 5 Experimental results

**Fig. 9:**
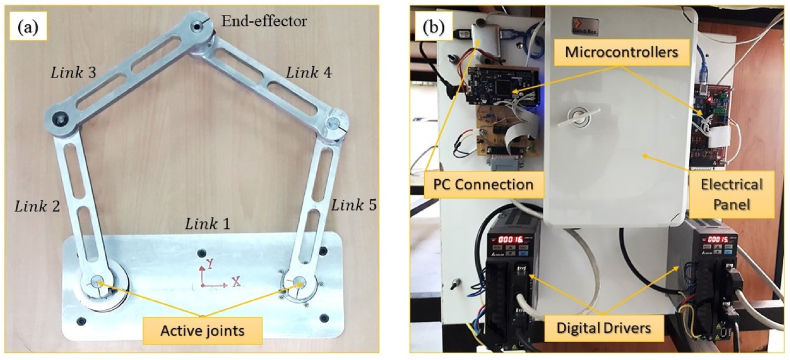
Experimental platform of 5-bar robot. (a) The top view of 5-bar parallel manipulator. (b) The driving and control system of ZNU Five-bar robot consists of two digital drivers, two microcontroller boards, and electrical panel.

In this section, the experimental results on a 5-bar testbed are presented. The 5-bar setup is illustrated in Fig. 9. The robot is actuated by two motors which are connected to the microcontrollers through two digital drivers. The so-called drivers collect the joint positions and send the command torques to the actuators. The maximum torque of the motors is $u_{\max}=1.274\;N.m$. The controllers are commanded from a personal computer (PC) and also send the outputs to the PC during the control process. The sample time of the signals is 0.01 s. The robot arms are made of 7000 series aluminum alloy and their specifications are listed as follows: $m_{2,5}=448\;gr$, $m_{3,4}=495\;gr$, $L_{1}=310\;mm$, $L_{2,3,4,5}=250\;mm$, $I_{3,4}=5.5082\times 10^{-3}kg.m^{2}$, $I_{2,5}=4.8205\times 10^{-3}kg.mm^{2}$, $J_{r}=0.2770\times 10^{-4}kg.m^{2}$ where $m_{i}$, $L_{i}$, $I_{i}$ and $J_{r}$ represent the mass, length, mass moment of inertia of i ^th^ link, and rotor inertia, respectively.

In the experiment, the reference signals are defined as task space trajectory, which are applied to a 5-bar robot as: $x=-rsin\left(\left(2\pi f\right)t+\varphi_{0}\right)+x_{1}$ and $y=-rsin\left(\left(2\pi f\right)t+\varphi_{0}\right)+y_{1}$ where r=50.00mm, f=0.50, $\varphi_{0}=\frac{\pi }{3}\;rad$, $x_{1}=0$ and $y_{1}=446.15\;mm$. The parameters of the proposed controller (33) are determined using the genetic algorithm approach and fitness function (44), and are illustrated in Table 2.

**Fig. 10:**
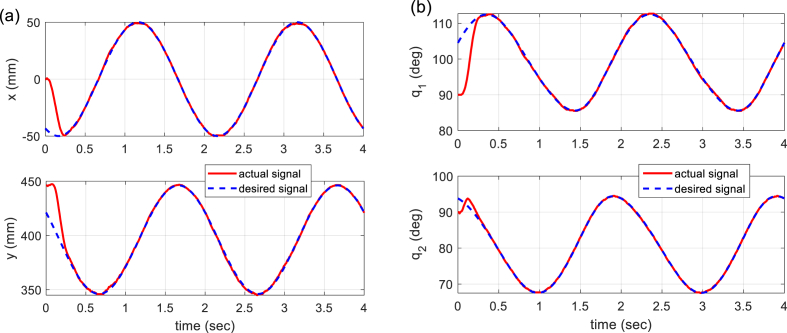
Experimental results of trajectory tracking. (a) Time history of end-effector position in task space (*x*, *y*). (b) Time history of active joints angular position in joint space (*q*_1_, *q*_2_).

**Fig. 11:**
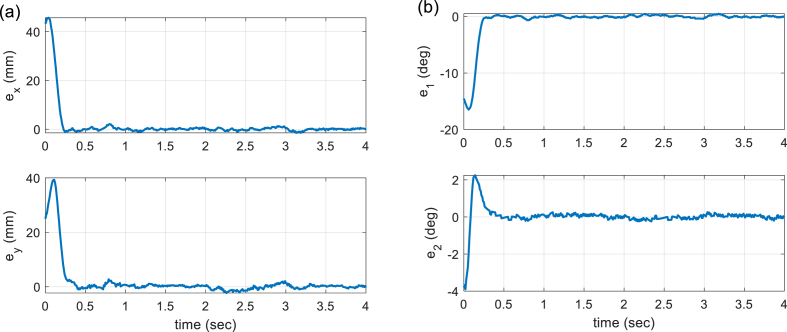
Tracking error signals. (a) Time history of end-effector tracking errors (*e*_*x*_, *e*_*y*_). (b) Time history of active joints tracking errors (*e*_1_, *e*_2_).

**Fig. 12:**
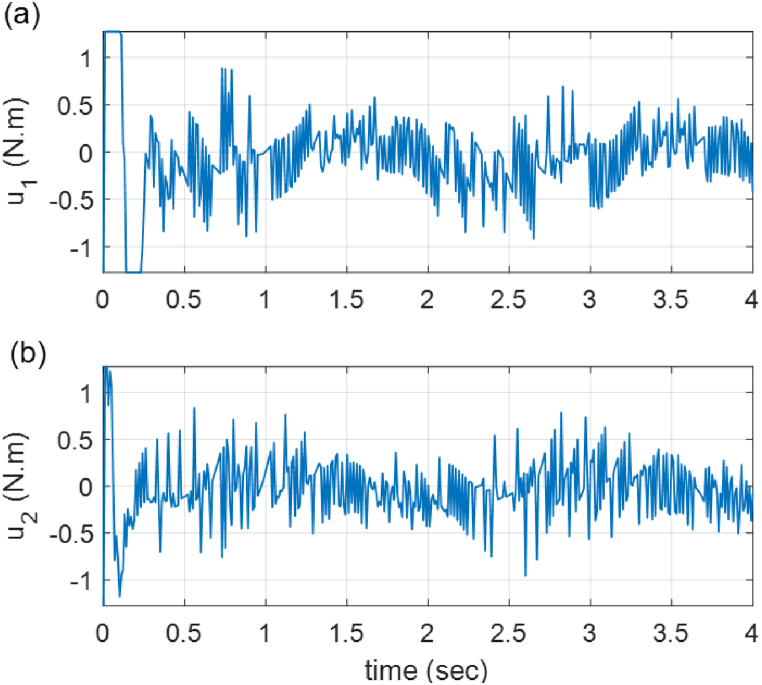
Time histories of the experimental control signals. (a) *u*_1_, (b) *u*_2_.

The experimental results on the 5-bar parallel robot are illustrated in Fig. 10, Fig. 11, Fig. 12.

Fig. 10 provides the time histories of the end-effector position in both task and joint space. It shows that the tracking action is fulfilled under 0.4 (sec). In order to show more clearly the tracking performance, Cartesian error signals, as well as joint errors, are given in Fig. 11. Finally, Fig. 12 illustrates the control signals generated by the proposed controller to command the robot actuators. A video of the performance of the built 5-bar parallel robot is available at https://youtu.be/xPyZbsL1xFk.

## 6 Conclusions

This paper proposed an optimal adaptive barrier-function super-twisting sliding mode control scheme based on genetic algorithms and global nonlinear sliding surface for the trajectory tracking control of parallel robots in the presence of uncertainties and external disturbances. Attributes of the proposed approach are its global property, which eliminates the reaching phase thereby guaranteeing system stability from the initial state and the elimination of the requirement of availability of information about the upper bounds of external disturbances; which makes it more realistic for practical implementation. The proposed approach was assessed using both a simulation study and a practical implementation. The results were further compared to that of an ANSMC and PID controllers. The simulation results showed that the tracking error reached zero in less than 1.5 s, when using the proposed approach and 3 s for the ANSMC controller, whereas the error never converged to zero when using the PID controller. The experimental results showed that the error reached its minimum range in less than 0.5 s and the robot was able to perfectly follow the commanded trajectory. Our future research directions will focus on including a fault tolerant control component to mitigate faults such as loss of actuator effectiveness, lock-in-place and float faults. We will also investigate extending the proposed approach to the control of parallel robots with flexible links.

## Author contribution statement

Mostafa Barghandan: Conceived and designed the experiments; Performed the experiments; Analyzed and interpreted the data; Contributed reagents, materials, analysis tools or data; Wrote the paper.

Ali Akbar Pirmohamadi: Conceived and designed the experiments; Contributed reagents, materials, analysis tools or data; Wrote the paper.

Saleh Mobayen, PhD: Analyzed and interpreted the data; Contributed reagents, materials, analysis tools or data; Wrote the paper.

Afef Fekih: Analyzed and interpreted the data; Wrote the paper.

## Funding statement

This research did not receive any specific grant from funding agencies in the public, commercial, or not-for-profit sectors.

## Data availability statement

No data was used for the research described in the article.

## Declaration of interest’s statement

The authors declare no competing interests.

## Figures and Tables

**Table 3:** Geometry and Static characteristics of the Stewart Manipulator.

Symbol	Description	Value	Unit
m	Mass of the moving platform	34.6	kg
$I_{x},I_{y}$	The moving platform mass moment of inertia about X, Y, Z	0.4587	$kg.m^{2}$
$I_{z}$		0.9123	
$r_{b}$	The base plate surrounded circle radius	300	mm
$r_{p}$	The Moving platform surrounded circle radius	200	
l	The legs reference length	900	
$\begin{array}{c} P_{1}\\ \vdots \\ P_{6} \end{array}$	The joints position of the upper platform w.r.t moving frame	{193.18, 51.76, 0}	mm
		{-51.76, 193.18, 0}	
		{-141.42, 141.42, 0}	
		{-141.42, −141.42, 0}	
		{-51.76, −193.18, 0}	
		{193.18, −51.76, 0}	
$\begin{array}{c} B_{1}\\ \vdots \\ B_{6} \end{array}$	The joints position of the lower platform w.r.t inertia frame	{192.83, 229.81, 0}	mm
		{102.60, 281.90, 0}	
		{-295.44, 52.09, 0}	
		{-295.44, −52.09, 0}	
		{102.60, −281.90, 0}	
		{192.83, −229.81, 0}	

## Appendix A Computation of the SM nominal matrices

(45) $$M_{0}=\left[\begin{array}{cccccc} m & 0 & 0 & 0 & 0 & 0\\ 0 & m & 0 & 0 & 0 & 0\\ 0 & 0 & m & 0 & 0 & 0\\ 0 & 0 & 0 & M_{44} & M_{45} & M_{46}\\ 0 & 0 & 0 & M_{54} & M_{55} & M_{56}\\ 0 & 0 & 0 & M_{64} & M_{55} & M_{66} \end{array}\right]\text{,}$$

$$M_{44}=I_{X}\;\cos^{2}\;\gamma +I_{Y}\;\sin^{2}\;\gamma \;M_{45}=\left(I_{X}-I_{Y}\right)\cos\;\alpha \;\cos\;\gamma \;\sin\;\gamma M_{46}=0,M_{54}=M_{45}\;M_{55}=I_{X}\;\cos^{2}\;\alpha \sin^{2}\;\gamma +I_{Y}\;\cos^{2}\;\alpha \cos^{2}\;\gamma +I_{Z}\;\sin^{2}\;\alpha M_{56}=-I_{Z}\;\sin\;\alpha \;M_{64}=M_{46},M_{65}=M_{56},M_{66}=I_{Z}$$

(46) $$V_{0}=\left[\begin{array}{cccccc} 0 & 0 & 0 & 0 & 0 & 0\\ 0 & 0 & 0 & 0 & 0 & 0\\ 0 & 0 & 0 & 0 & 0 & 0\\ 0 & 0 & 0 & V_{44} & V_{45} & V_{46}\\ 0 & 0 & 0 & V_{54} & V_{55} & V_{56}\\ 0 & 0 & 0 & V_{64} & V_{65} & V_{66} \end{array}\right]\text{,}$$

$$V_{44}=-\frac{1}{2}\left(I_{X}-I_{Y}\right)\sin\left(2\gamma \right)\dot{\gamma }$$

$$V_{45}=+\frac{1}{2}\left(I_{X}+I_{Y}-2I_{Z}\right)\cos\left(\alpha \right)\sin\left(\alpha \right)\dot{\beta }+\frac{1}{4}\left(I_{Y}-I_{X}\right)\cos\left(\alpha \right)\left(\sin\left(\alpha -2\gamma \right)-\sin\left(\alpha +2\gamma \right)\right)\dot{\beta }+\frac{1}{2}I_{Z}\;\cos\left(\alpha \right)\dot{\gamma }+\frac{1}{2}\left(I_{X}-I_{Y}\right)\cos\left(\alpha \right)\cos\left(2\gamma \right)\dot{\gamma }$$

$$V_{46}=\frac{1}{2}\left(I_{Y}-I_{X}\right)\sin\left(2\gamma \right)\dot{\alpha }+\frac{1}{2}I_{Z}\;\cos\left(\alpha \right)\dot{\beta }+\frac{1}{2}\left(I_{X}-I_{Y}\right)\cos\left(\alpha \right)\cos\left(2\gamma \right)\dot{\beta }$$

$$V_{54}=\frac{1}{2}\left(I_{Y}-I_{X}\right)\sin\left(\alpha \right)\sin\left(2\gamma \right)\dot{\alpha }+\frac{1}{4}\left(2I_{Z}-I_{X}-I_{Y}+\left(I_{X}-I_{Y}\right)\cos\left(2\gamma \right)\right)\sin\left(2\alpha \right)\dot{\beta }+\frac{1}{2}I_{Z}\;\cos\left(\alpha \right)\dot{\gamma }+\frac{1}{2}\left(I_{X}-I_{Y}\right)\cos\left(\alpha \right){\cos\left(\gamma \right)}^{2}\dot{\gamma }-\frac{1}{2}\left(I_{X}-I_{Y}\right)\cos\left(\alpha \right){\sin\left(\gamma \right)}^{2}\dot{\gamma }$$

$$V_{55}=\frac{1}{2}I_{Z}\;\sin\left(2\gamma \right)\dot{\alpha }-\frac{1}{2}\left(I_{Y}{\cos\left(\gamma \right)}^{2}+I_{X}{\sin\left(\gamma \right)}^{2}\right)\sin\left(2\gamma \right)\dot{\alpha }+\frac{1}{2}\left(I_{X}-I_{Y}\right){\cos\left(\alpha \right)}^{2}\;\sin\left(2\gamma \right)\dot{\alpha }$$

$$V_{56}=\frac{-1}{2}I_{Z}\;\cos\left(\alpha \right)\dot{\alpha }+\frac{1}{2}\left(I_{X}-I_{Y}\right)\cos\left(\alpha \right)\cos\left(2\gamma \right)\dot{\gamma }+\frac{1}{2}\left(I_{Y}-I_{X}\right){\cos\left(\alpha \right)}^{2}\;\sin\left(2\gamma \right)\dot{\beta }$$

$$V_{64}=\frac{-1}{2}I_{Z}\;\cos\left(\alpha \right)\dot{\beta }+\frac{1}{2}\left(I_{X}-I_{Y}\right)\sin\left(2\gamma \right)\dot{\alpha }-\frac{1}{2}\left(I_{X}-I_{Y}\right)\cos\left(\alpha \right)\cos\left(2\gamma \right)\dot{\beta }$$

$$V_{65}=-\frac{1}{2}I_{Z}\;\cos\left(\alpha \right)\dot{\alpha }-\frac{1}{2}\left(I_{X}-I_{Y}\right)\cos\left(\alpha \right)\cos\left(2\gamma \right)\dot{\alpha }-\frac{1}{2}\left(I_{X}-I_{Y}\right){\cos\left(\alpha \right)}^{2}\;\sin\left(2\gamma \right)\dot{\beta }$$

$$V_{66}=0$$

(47) $$G_{0}\left(q\left(t\right)\right)={\left[0\;0\;mg\;0\;0\;0\right]}^{T}$$

where *m* is the mass of MP; $I_{X}$, $I_{Y}$, and $I_{Z}$ are mass moment of inertia of MP about *X, Y* and, *Z* axis; g is the gravitational acceleration;
